# Depressive Symptoms and Length of U.S. Residency Are Associated with Obesity among Low-Income Latina Mothers: A Cross-Sectional Analysis

**DOI:** 10.3390/ijerph14080869

**Published:** 2017-08-02

**Authors:** Ana Cristina Lindsay, Mary L. Greaney, Sherrie F. Wallington, Julie A. Wright, Anne T. Hunt

**Affiliations:** 1 Department of Exercise and Health Sciences, College of Nursing and Health Sciences, University of Massachusetts, Boston, MA 02125, USA; Julie.Wright@umb.edu; 2 Department of Nutrition, Harvard T.H. Chan School of Public Health, Boston, MA 02115, USA; 3 Health Studies and Department of Kinesiology, University of Rhode Island, Kingston, RI 02881, USA; mgreaney@uri.edu; 4 Lombardi Comprehensive Cancer Center, Georgetown University Medical Center, Washington, DC 20057, USA; slw49@georgetown.edu; 5 Hunt Consultants Associates; Chapel Hill, NC 27517 USA; hunt@post.harvard.edu

**Keywords:** depression, obesity, Latina, mothers, immigrant, low-income, United States, maternal health

## Abstract

Latinos are the largest minority population group in the United States (U.S.), and low-income Latina women are at elevated risk of depression and obesity. Thus, the prevention of these two problems is a pressing public health concern in this population. Both depressive symptoms and obesity are modifiable factors that can be addressed by culturally relevant interventions. However, the association between depressive symptoms and obesity in Latina immigrant women is not well understood. Therefore, this cross-sectional study examined the association between depressive symptoms and obesity among Latina women of childbearing age (15–44). Participants (*n* = 147) were low-income, predominantly immigrant Latina mothers enrolled in the Latina Mothers′ Child Feeding Practices and Style Study. Women were eligible to participate if they self-identified as Latina; were enrolled in or eligible for the Special Supplemental Nutrition Program for Women, Infants and Children program; had a child between ages two and five years; and were living in the U.S. for at least one year, and residing in Rhode Island. Enrolled participants completed a survey in their language of preference (English or Spanish) administered by bilingual interviewers. About one-third (34%) of participants were classified as having obesity (BMI ≥ 30 kg/m^2^), 28.3% had elevated depressive symptoms (CES-D ≥ 16), and 70.1% were immigrants. Women with elevated depressive symptoms had increased odds of having obesity (odds ratio (OR) = 2.80, 95% confidence interval (CI): 1.24–6.33). Additionally, among immigrants, length of U.S. residency was associated with increased odds of obesity (OR = 1.05, 95% CI: 1.02–1.09). Findings underscore the need for screening and culturally relevant interventions designed to address both depressive symptoms and obesity among low-income Latina women of childbearing age. Furthermore, findings highlight the importance of taking into account the length of residency in the U.S. when designing interventions targeting Latina immigrants.

## 1. Introduction

Latinos/Hispanics (hereafter referred to as Latino or Latina) are the largest and most rapidly growing minority population group in the United States (U.S.) [1] and are disproportionally affected by obesity and related chronic diseases [2]. Obesity is a significant public health problem among women of childbearing age (15–44 years of age) [2], with nearly two-thirds of women of childbearing age in the U.S. being overweight or obese [2]. Although obesity impacts all women of childbearing age, low-income Latina women are at elevated risk (42.5% Latina/Hispanic vs. 32.6% non-Hispanic) [2]. This disparity underscores the need to identify, understand, and address obesity risk factors amenable to culturally relevant interventions designed to meet the needs of low-income Latinas [2].

Prior research shows that obesity in women of childbearing age is associated with numerous short- and long-term adverse maternal and child health outcomes such as having a child with birth defects, having a child born with high or low birth weight, having a cesarean section, having increased risk of gestational diabetes, suffering from postpartum anemia, etc. [3,4,5,6,7,8,9]. Additionally, women with obesity are less likely to return to their pre-pregnancy weight, especially if weight gain has been above gestational weight gain recommendations [6,7,8,9,10]. Therefore, preventing and reducing obesity among women of childbearing age is an important public health goal, especially for Latina women who are disproportionally affected by this chronic disease [2].

Several risk factors, including acculturation, health literacy, and depression have been found by prior research to be associated with increased risk of obesity among minority women, including Latinas [11,12,13,14,15]. Acculturation, defined as the process of adopting cultural traits or social patterns of another group (in this case the U.S.), is a multidimensional, complex process including dimensions of language usage, migration status, generational status, duration of time spent in the country of migration, ethnicity of social networks, etc. [16,17,18]. Research evidence suggests that increasing acculturation is associated with poorer health outcomes including increased risk of obesity among immigrants to the U.S. [11,12,13]. Despite the range of constructs used to measure acculturation, the most consistent association between obesity and acculturation among Hispanics has been found with length of U.S. residency, with the longer duration (years) being associated with an increased risk of obesity [11,12,14,19]. Recent work by Lee et al. [13] suggests that increasing length of residency in the U.S. may result in the adoption of unhealthy behaviors, as well as greater exposure to harmful sources of psychosocial stress including racial and anti-immigrant discrimination. Prior research also suggests that the increased risk of obesity among immigrants is linked to the “acculturation process”, which includes, changes in diet (“dietary acculturation”), physical activity levels and increased exposure to an “obesogenic environment”, characterized by easy and increased access to relatively affordable high-density, low-nutrient value foods. These obesity-promoting factors contribute to energy imbalance and increased risk of obesity among immigrants [20,21,22,23,24,25]. 

Health literacy is an individual’s ability to access, understand, and apply basic health information to reduce his or her risks of a variety of preventable conditions and diseases [26,27,28,29,30,31,32]. Studies conducted in the U.S. have found that approximately 36% of adults have inadequate health literacy, and this number is higher for minority groups including Hispanics (65%) [26]. This is a concern as inadequate or low health literacy serves as a barrier to individuals understanding health information and taking adequate action to reduce their risk for various preventable conditions and diseases, including obesity [30,31,32]. For example, research shows that inadequate health literacy is negatively associated with several health promoting behaviors [23,29], such as physical activity, healthy eating [27], use of health care [30], etc., which, in turn, have been linked to increased risk of obesity. 

Increasingly, research documents the link between depression and obesity among women of childbearing age, and these findings suggest the importance of addressing depression among women in this age group [6,11,12,13,14]. Depression among women of childbearing age is associated with negative consequences for the women themselves (e.g., inadequate prenatal care, poor nutrition, higher preterm birth, low birth weight, preeclampsia, and spontaneous abortion), their children (e.g., socio-emotional, cognitive, and behavioral problems), and their families (e.g., negative changes in family communication, nurturance, activities and routines, and cohesion) [7,8,9,10]. According to the National Institute of Mental Health, one in seven women of reproductive age in the U.S. are affected by depression, and 15% experience postpartum depression [5].

Recent research indicates that the prevalence of depression among U.S.-born and foreign-born Latinos ranges from 22.3% to 38.0% [19]. In addition, studies suggest that length of residency in the U.S. is associated with increased risk of depression [7,8,9,10,15,33,34,35,36,37,38,39,40]. For example, a recent study documented that Latina immigrants who had lived in the U.S. for at least 15 years had higher depressive symptoms than U.S.-born Latinas [19]. Researchers have suggested that the process of acculturation, which encompasses psychological, physical and social difficulties in the transition and adaptation to a new culture, may explain this increase in depressive symptoms among immigrants, what has been referred to as “acculturative stress” [41].

Given that both depression and obesity are prevalent among Latina women of childbearing age and have significant negative health outcomes for mothers and children, it is important to understand the relationship between depression and obesity so appropriate screening routines and interventions can be developed to lessen the negative health consequences for both mothers and children [42,43,44]. Although research examining the associations between depression and obesity among women of childbearing age is increasing [10,45,46,47,48], there is still a dearth of studies on low-income Latina mothers [9,46,47]. Additional research is needed since results of currently available studies have not been consistent—some studies have documented significant associations between depression and obesity [9,46,47,48,49] whereas others have found no significant associations [43]. Therefore, the primary purpose of this study was to expand on the existing literature by exploring the association between maternal depressive symptoms and risk of obesity among a sample of primarily immigrant, low-income Latina women of childbearing age, while taking into account the potential effects of acculturation, health literacy, and demographic characteristics.

## 2. Materials and Methods 

### 2.1. Study Design, Setting and Sample

This study is a secondary analysis of data collected as part of the Latina Mothers Child Feeding Practices and Styles (LMCFPS) Study, a cross-sectional study conducted in Rhode Island, U.S. between April 2015 and June 2016. The LMCFPS study examined psychosocial and cultural influences on child feeding styles and practices of low-income Latina mothers of preschool-aged children enrolled in or eligible for the Special Supplemental Nutrition Program for Women, Infants and Children (WIC, household income ≤185% of the poverty level). 

### 2.2. Ethics, Consent, and Permissions

Study participants were recruited from local agencies and community-based health and social programs. Women were eligible to participate in the study if they: (a) self-identified as Latina; (b) were ≥18 years of age; (c) had at least one child aged 2–5 years; (d) were participating in WIC or were WIC eligible; (e) lived in Rhode Island; and (f) resided in the U.S. for at least one year. Prior to enrolling in the study, eligible women were read the consent forms in their preferred language (English or Spanish) by trained bilingual, bicultural interviewers. After providing a signed informed consent form, women completed an interviewer-administered survey in their preferred language. The study protocol was approved by the Institutional Review Board of the University of Massachusetts–Boston. 

### 2.3. Measures 

#### 2.3.1. Depressive Symptoms

Maternal depressive symptoms were measured using the Center for Epidemiological Studies Depression Scale (CES-D), a non-diagnostic questionnaire, which is a valid and reliable screening tool for use in non-clinical samples [50]. The CES-D has been validated with multiethnic samples [51,52] and widely used with Latino populations [46,47]. Additionally, the CES-D has undergone reliability and validity testing among multiple ethnic groups, with Latinos in the U.S. and internationally reporting high Cronbach′s alphas (0.85–0.88) [53,54]. It includes 20 items that ask participants to report the number of days that they experienced specific symptoms such as being fearful or not being able to shake off the blues over the past week. Responses are scored and then summed, with summary scores ranging from 0 to 60. Summary scores are dichotomized, with a 16 or higher indicating possible risk for clinically defined depressive disorders and need for further evaluation [33,49].

#### 2.3.2. Weight Status

Maternal body mass index (BMI) was calculated from participants′ self-reported height and weight and used to calculate weight status. Participants were dichotomized as having obesity (BMI ≥ 30 kg/m^2^) or non-obese (BMI < 30 kg/m^2^) based on associations with disease risk [2]. 

#### 2.3.3. Covariates

A number of covariates were examined based on the relevant literature, including the following:

##### Acculturation Level

Acculturation was measured using the Short Acculturation Scale for Hispanics (SASH), a 12-item scale validated for use in Latino populations [16,17]. The SASH assesses language use, media use, and ethnic social relations, and has good reliability (Cronbach’s alpha reliabilities 0.92–0.89 for the overall SASH scale, 0.89 for language use, 0.88 for media preference, and 0.72 for ethnic and social relations) [16,18]. 

An acculturation score was computed for each participant by averaging across 12 items, measured on a scale of 1 to 5. Low acculturation was defined by the scale developers as having a mean SASH score <2.99 and used in the present study as a dichotomized score (high: ≥2.99; low: <2.99) [35]. In addition, length of residence in the U.S. measured in years was assessed as an indicator of acculturation [16,18]. 

##### Health Literacy Level

Health literacy was measured using the Spanish version of the Short Test of Functional Health Literacy in Adults (S-TOFHLA) [55], a frequently used 36-item instrument that assesses reading comprehension and numeracy [27,56]. The S-TOFHLA has been validated (Cronbach′s alpha = 0.80) for use in English and Spanish [55]. Health literacy was categorized as inadequate (score 0–16), marginal (17–22), or adequate (23–36). For analysis and to be comparable to other studies, health literacy was dichotomized a priori (high literacy: S-TOFHLA ≥ 23, and low literacy: S-TOFHLA < 23) [57].

##### Demographics

Participants reported their age, country of origin, primary language, educational attainment (≥high school graduate/general education degree (GED) vs. < high school/GED), and family annual income (≥US$30,000/year vs. <US$30,000/year).

### 2.4. Data Analysis

All analyses were conducted using SAS 9.4 [58]. Descriptive statistics were calculated for all key variables and differences assessed in demographics by depressive symptoms (CES-D ≥ 16: high; CES-D < 16: Low) using means and standard deviations for continuous variables and frequencies and percent for categorical variable. Chi-square and Fisher′s exact tests were used to determine if there were differences between depression status groups by key demographic variables. Although we collected information on participants′ country of origin, the small sample sizes in the various groups only allowed this information to be used for descriptive purposes. The relationship between depressive symptoms and weight status then was assessed using logistic regression. Odds ratios (ORs) for obesity were calculated for mothers classified as having elevated depressive symptoms (CES-D score ≥ 16). All models controlled for mothers′ age, income, educational attainment, length of U.S. residence, acculturation, and health literacy, which were assessed for multicollinearity prior to inclusion. Backward elimination was used to identify the final, most parsimonious model.

## 3. Results

### 3.1. Participants 

The LMCFPS study sample included 208 mothers, 61 of whom did not report height and weight data, resulting in an analytic sample of 147 participants (71% of total sample). Women who reported height and weight did not differ from those who did not by basic socio-demographics (e.g., age, education, income, etc.).

#### Participants′ Demographic, Psychosocial, and Cultural Characteristics

Mothers had a mean age of 32 years old (SD = 6.31; range 19–50), two-thirds (68%) had graduated from high school or earned a GED, and the majority (84%) had family incomes of less than $30,000/year, which is below the federal poverty line (see Table 1). Approximately 70% of the mothers were born outside the U.S. Of the mothers born outside the U.S., 83% were categorized as having low acculturation levels (SASH < 2.99) and the mean length of U.S. residence was 11.8 years (SD = 7.6; range 1–40). Spanish was the primary language for all (100%) mothers, both U.S. and foreign-born, and 85% of participants were categorized as having low health literacy (S-TOFHLA < 23).

### 3.2. Elevated Depressive Symptoms 

About half (51%) of mothers with elevated depression scores (CES-D ≥ 16) also had obesity compared to 28% of mothers with low CES-D (<16) scores (X^2(1)^ = 6.52, *p* < 0.01). There was no difference in presence of depressive symptom status by maternal age, education, income, born in the U.S., years of residence in the U.S., acculturation level and health literacy scores (see Table 2).

### 3.3. Correlates of Obesity 

In the final adjusted logistic regression, mothers who were classified as having elevated depressive symptoms were 2.8 times (OR = 2.80, 95% CI: 1.24–6.33) more likely to be obese than mothers who were classified as having low depressive symptoms (see Table 3). Additionally, length of U.S. residency was a significant covariate (OR = 1.05, 95% CI: 1.02–1.09), with the odds of having obesity increasing by 5% for each year living in the U.S., regardless of maternal depression status. None of the other examined covariates were significant (see Table 3). 

## 4. Discussion

This study found that elevated depressive symptoms and obesity were prevalent in this sample of low-income, predominantly immigrant Latina mothers: 28.3% of participants had elevated depression symptoms and 34% had obesity. Study findings are within the range reported by previous studies in this population [47,48,59,60]. Prior studies have reported obesity prevalence rates ranging from 17.9% to 44.4% [47,48] and prevalence of elevated depressive symptoms ranging from 11.1% to 32.4% when assessed using diverse instruments and cut-off points [21,42,43,61], as well as the same instrument and cut-off points used in this study [46,48,61,62]. 

In addition, study findings indicate that length of residency in the U.S. was associated with increased odds of maternal obesity for immigrant Latina mothers. This finding is important and aligns with results of previous research documenting that length of residence in the U.S. is associated with increased risk of becoming overweight or obese among immigrants [19,63,64,65,66]. Research suggests that the longer one lives in the U.S., the greater obesity risk due to changes in diet, physical activity levels, increased stress, cultural norms, and reduced social networks, all of which may adversely affect weight status [11,19]. A recent study [11] examining data from the National Latino and Asian American Survey, and using path analytic methods to assess duration of stay in the U.S. and BMI among Latino and Asian immigrants, found that for Latina women, acculturative stress is a significant indirect pathway to explaining the effects of the duration of stay in the U.S. on BMI. Our findings suggest that interventions should consider the negative impact that length of residency in the U.S. has on risk of obesity among Latina immigrants. 

Notably, acculturation level was not associated with risk of obesity in this study. This finding concurs with that of some previous research conducted with Latinas that has documented that it is years of residency in the U.S., and not necessarily language acculturation, that increases Latino immigrants’ risk of obesity [19,67]. Mounting research evidence points to the complex nature of disentangling the effects of acculturation on health outcomes including obesity among Latino immigrants [11,12,13,14,68]. The cross-sectional design of our study does not allow for examination of the complex effects of acculturation on depressive symptoms and obesity. Nonetheless, further research is needed given the public health significance of both mental health and obesity to health disparities among Latinos, and the evidence of associations between acculturation level and risk of obesity and depressive symptoms. Future studies should explore the association and mechanisms between maternal depression and aspects of immigration that may affect Latina mothers′ risk of obesity and vice-versa.

Moreover, the process of acculturation has been linked to both increased risk of depression and obesity among racial and ethnic minority immigrants, including Latinas [11,12,13,14,19,42,43,44,45,69,70]. Mounting evidence suggests that the acculturation process leads to “acculturation stress”, which may be due to a range of factors including low socio-economic status and income inequality, low educational levels, separation from family members including children, lack of social support, language barriers, lack of access to healthcare, and marginalization/isolation, fear of deportation [11,12,13,14,42,43,44,45]. The acculturation process also involves transition to a new food environment, including exposure to and increased access to and availability of relatively inexpensive high-density foods, and decreased levels of physical activity, which contribute to an increased risk of obesity among Latinos [20,21,22,23,24,69,70].

Study findings revealed a bi-directional relationship between maternal depressive symptoms and obesity, which has been documented previously [4,10,45]. These findings suggest the importance of addressing depression and obesity simultaneously. Given that both maternal depression and obesity are modifiable factors associated with adverse maternal and child outcomes, this finding underscores the need for assessing and addressing maternal mental health in the pre- and post-natal periods [8,35]. Interventions designed to reduce depressive symptoms are likely to contribute to reduction in risk of obesity and vice-versa, interventions targeted at prevention and control of obesity are likely to contribute to a reduction in the symptoms of depression [40,46]. 

Finally, although we did not find an association between health literacy and obesity, it is important to note that 85% of study participants were categorized as having low health literacy (S-TOFHLA < 23). Therefore, interventions designed to address both mental health issues including depressive symptoms and obesity in low-income Latina immigrants should take into account the low health literacy levels of this population.

Study findings should be considered in light of several limitations. First, maternal BMI was calculated using self-reported data, which may underestimate BMI and thus bias the findings in unknown ways [71,72]. Additionally, only 71% of the LMCFPS study sample reported their height and weight, and this missing data may not be random. Nevertheless, when comparing the depressive symptom scores of mothers who reported height and weight and those who did not, we did not find a significant difference. Furthermore, the relatively small sample size and limited power may have limited the ability to assess the association of covariates (e.g., maternal age, income, education level, and health literacy) that have been previously reported to be associated with risk of obesity in women of childbearing age. In addition, the small and unique study sample limits the generalizability of study findings. Finally, given the cross-sectional design neither temporality, nor bi-directionality of the relationship between depressive symptoms and obesity can be determined. Study strengths include a multiethnic sample of Latina mothers and the use of more than one measure of acculturation (SASH and length of residence).

## 5. Conclusions

Given the disproportionate risk of obesity and depressive symptoms among low-income, immigrant Latina women of childbearing age [3,8,9,10,11,12,13,14,46,47,48,59], the findings of the present study indicate that there is a need to address both maternal depression and obesity in low-income Latina immigrant mothers. Study results add to the current literature and can inform the design of interventions developed to prevent and control obesity targeting low-income Latina women of childbearing age. Interventions culturally tailored to meet the needs of low-income Latina mothers may reduce the multiple adverse health effects of maternal depression among this high-risk population group. Previous research conducted with Latinos indicates the importance of addressing and incorporating sociocultural influences, such as family separation, acculturative stress, racism and discrimination, and poverty, on maternal depression by building upon the family and cultural strengths and assets, including family cohesion, nuclear and extended family involvement, cultural traditions, bicultural orientation, and community supports [38,44]. Given the public health significance of both depression and obesity, and the potential adverse effects that both obesity and depressive symptoms have on maternal and child health, interventions targeting Latina women of childbearing age are likely to have positive effects on the health outcomes of both Latina women and their children.

## Figures and Tables

**Table 1:** Characteristics of study the sample (*n* = 147).

Socio-demographic Variables	Categories	*n*	% or Mean (SD)
**Age (years)**		89	32 (SD 6.31)
**Education**			
	Less than high school or GED ^1^	47	32.0
	High School-GED or higher	100	68.0
**Income**			
	<30,000/year	84	80.0
	≥30,000/year	21	20.0
**Born outside the U.S.**			
	Yes	103	70.1
	No	44	29.9
**Country of Origin**			
	Guatemala	36	35.5
	Dominican Republic	21	20.8
	Colombia	15	15.1
	Mexico	14	13.9
	El Salvador	9	8.9
	Peru	3	3.1
	Venezuela	1	0.9
	Cuba	1	0.9
	Uruguay	1	0.9
**Acculturation variables**			
Years of residence in the U.S.		103	(11.8, SD 7.6 years)
SASH ^2^ score			
	High	25	17.0
	Low	122	83.0
**Health Literacy** S-TOFHLA^3^ score			
	High ≥ 23	22	15.0
	Low < 23	125	85.0
**Obesity status ^4^**			
	Obese (BMI ≥ 30.0 kg/m^2^)	50	34.0
	Non-obese (BMI < 30.0 kg/m^2^)	97	66.0
**Depression (CES-D) ^5^**			
	CES-D < 16	99	71.7
	CES-D ≥ 16	39	28.3

^1^ GED: General Education Degree; ^2^ SASH: Short Acculturation Scale for Hispanics; ^3^ S-TOFHLA: Short Test of Functional Health Literacy in Adults; ^4^ BMI: Body Mass Index; ^5^ CES-D: Center for Epidemiologic Studies Depression Scale.

**Table 2:** Prevalence of elevated depressive symptoms by CES-D cut-off scores and selected variables among low-income Latina mothers (*n* = 147).

Socio-Demographic Variables	CES-D < 16			CES-D ≥ 16			*p*-Value
	(*n* = 99)			(*n* = 39)			
	*N*	%	M (SD)	*n*	%	M (SD)	
**Age**	99	71.7	31.5 (5.5)	39	28.3	32.3 (7.9)	0.54
**Education**							
<high school/GED	29	21.0		13	9.4		0.64
≥High School/GED	70	50.7		26	18.8		
**Income**							
<$30,000/year	59	60.8		18	18.6		0.54
≥$30,000/year	14	14.4		6	6.2		
**Born in U.S.**							
Yes	31	22.5		11	20.3		0.72
No	68	49.3		28	8.0		
**Acculturation variables**							
**Years of residence in the U.S.**	98	72.6	17.0 (11.3)	37	27.4	17.3 (11.1)	0.88
**SASH score**							
High	17	12.3		6	4.4		0.80
Low	82	59.4		33	23.9		
**Health Literacy variable**							
**S-TOFHLA**							
High ≥ 23	16	11.6		5	3.6		0.62
Low < 23	83	60.1		34	24.6		
**Obesity status**							
Obese (BMI ≥ 30.0)	28	20.3		20	14.5		0.01
Non-obese (BMI < 30.0)	71	51.5		19	13.8		

**Table 3:** Results from multivariable logistic regression models estimating odds of maternal obesity associated with maternal depressive symptoms, controlling for covariates in low-income Latina mothers (*n* = 147).

Outcome: Obesity Status (Obese/Non-Obese)						
	Model 1 OR (CI) *	Model 2 OR (CI)	Model 3 OR (CI)	Model 4 OR (CI)	Model 5 OR (CI)	Model 6 OR (CI)
**Maternal age (years)**	1.00 (0.90, 1.12)					
**Income**	2.28 (0.39, 13.36)	0.99 (0.31, 3.09)				
<$30,000/year vs. ≥$30,000/year						
**Education**	0.33 (0.06, 1.66)	0.64 (0.22, 1.87)	0.90 (0.37, 2.17)			
≥HS/GED vs. <HS/GED						
**Health literacy (S-TOFHLA)**	1.92 (0.17, 21.55)	1.92 (0.43, 8.55)	1.77 (0.57, 5.52)	1.83 (0.61, 5.50)		
Adequate vs. Inadequate						
**Acculturation (SASH)**	0.34 (0.05, 2.23)	0.56 (0.17, 1.88)	0.44 (0.14, 1.36)	0.43 (0.14, 1.31)	0.43 (0.14, 1.31)	
High vs. Low						
**Years in the U.S.**	**1.11 (1.03, 1.20)**	**1.04 (1.00, 1.09)**	**1.07 (1.03, 1.12)**	**1.07 (1.03, 1.12)**	**1.07 (1.03, 1.11)**	**1.05 (1.02, 1.09)**
**Maternal depression**	**5.32 (1.10, 25.90)**	**2.66 (0.99, 7.17)**	**2.83 (1.24, 6.47)**	**2.84 (1.25, 6.48)**	**2.80 (1.23, 6.35)**	**2.80 (1.24, 6.33)**
CES-D ≥ 16 vs. CES-D < 16						

* 95% confidence interval; Boldface indicates *p*-value < 0.05.

## Abbreviations

The following abbreviations are used in this manuscript:

BMI	Body Mass Index
CES-D	Center for Epidemiologic Studies Depression Scale
CI	Confidence Interval
GED	General Education Degree
LMCFPS	Latina Mothers Child Feeding Practices and Styles
OR	Odds Ratio
SASH	Short Acculturation Scale for Hispanics
S-TOFHLA	Short Test of Functional Health Literacy in Adults
U.S.	United States
WIC	Special Supplemental Nutrition Program for Women, Infants and Children
